# Self-expanding metal stent insertion by colorectal surgeons using a two-person approach colonoscopy without fluoroscopic monitoring in the management of acute colorectal obstruction: a 14-year experience

**DOI:** 10.1186/s12957-021-02309-z

**Published:** 2021-07-02

**Authors:** Fei-hu Yan, Yao Zhang, Cheng-ling Bian, Xiao-shuang Liu, Bing-chen Chen, Zhen Wang, Hao Wang, E. Ji-fu, En-da Yu

**Affiliations:** 1 Department of Colorectal Surgery, Changhai Hospital, Naval Military Medical University, Shanghai, 200433 China; 2 Department of Medical Statistics Faculty of Medical Services, PLA Navy Medical University, 200433 Shanghai, China; 3 Department of Gastroenterology, Ruijin Hospital, Shanghai JiaoTong University School of Medicine, Shanghai, 200025 China; 4 Department of Radiology, Changhai Hospital, Naval Military Medical University, Shanghai, 200433 China; 5 Department of General Surgery, Shuguang Hospital, Shanghai University of Traditional Chinese Medicine, Shanghai, 201203 China; 6 Department of General Surgery, Eastern Theater Naval Hospital, Zhoushan, 316000 China

**Keywords:** Colorectal stenting, No fluoroscopy guiding, Large bowel obstruction, Colorectal cancer, Extra-luminal compression, Benign colorectal diseases, Recurrent tumor, Learning curve

## Abstract

**Background:**

Placement of a self-expanding metal stent (SEMS) in patients presenting with an acute colorectal obstruction (ACO) may obviate emergency surgery (ES), potentially effectively palliating incurable tumors, acting as a bridge to surgery (BTS) in patients with operable or potentially operable tumors and achieving effective decompression of other ACO. We present our experience with SEMS insertion by colorectal surgeons without fluoroscopic monitoring for ACO especially for acute malignant colorectal obstruction (AMCO) for nearly a 14-year period (2007–2020).

**Aim:**

To explore the safety and effectiveness of SEMS insertion in the management of ACO by colorectal surgeons using a two-person approach colonoscopy without fluoroscopic monitoring.

**Methods:**

We reviewed the medical records of patients retrospectively to identify all patients presenting to our unit with ACO especially with AMCO who had stenting carried out to achieve colonic decompression. All 434 procedures were performed by colorectal surgeons using a two-person approach colonoscopy without fluoroscopic monitoring.

**Results:**

The overall technique success rate and clinic success rate by SEMS insertion were 428/434 (98.6%) and 412/434 (94.9%). The overall incidence of complications by SEMS insertion was 19/434 (4.4%). The complications included clinical perforation (6/434, 1.4%); stent migration (2/434, 0.5%), 1 of which re-stent; stent detachment (fell off) (3/434, 0.7%), none of them with re-stent; stool impaction (6/434, 1.4%), 1 of which re-stent; and abdominal or anal pain (2/434, 0.5%). There was no hemorrhage in any of the 434 patients.

**Conclusions:**

SEMS insertion is a relatively safe and effective technique for colonic decompression in dealing with ACO as either a BTS or as a palliative measure. It is also a solution to other causes of ACO such as recurrent tumor, benign diseases, or extra-luminal compression. Therefore, ES was largely avoided.

## What does this paper add to the literature?

This study provides a successful decompression experience of self-expanding metal stent (SEMS) insertion in the management of acute colorectal obstruction (ACO), using a two-person approach colonoscopy without fluoroscopic monitoring.

## Background

Colorectal cancer is one of the most common cancer, approximately 10–20% of the patients with colorectal cancer present with large bowel obstruction, and those who present with ACO require urgent decompression because it can cause electrolytic fluid imbalance, colonic necrosis, bacterial translocation, and even death [1]. Placement of SEMS for ACO is a major endoscopic treatment that has been available since1991 [2]. The placement of colonic SEMS for palliation or BTS has been increasingly reported in the past 30 years. Substantial concerns of tumor seeding following SEMS placement, especially in case of perforation, have been raised in numerous studies. Actually, no significant differences are reported in oncologic long-term survival between patients undergoing stent placement as a BTS and those undergoing ES [3, 4]. ES is associated with several disadvantages such as increased postoperative morbidity and mortality rates [5], higher stoma rate, and lower curative resection rate than elective resection [6]. The use of SEMS to restore luminal patency is a more reasonable alternative to ES in acute malignant colorectal obstruction (AMCO). It can better avoid the risks and disadvantages associated with ES. Relieving the ACO in this approach has the following advantages: (1) stenting essentially makes the surgery more like an elective one-stage procedure, reducing the temporary stoma need [7]; (2) it serves as a definitive palliative treatment in patients unfit for surgery, especially who have disseminated, metastatic disease near the end of life [8]; (3) effective decompression enables full oncologic staging, which can help patients to choose suitable further treatment [9]; (4) effective decompression can provide opportunity and time to correct metabolic disturbances and malnutrition; (5) it makes neoadjuvant therapy without surgical intervention possible; (6) it is an effective approach in extra-luminal tumors causing ACO [10]; and (7) it is a solution to other ACO such as recurrent tumor and benign diseases.

With the recommendation of stents and the current clinical practice situation, it has been gradually used as one of the preferred intestinal decompression schemes. Although recent studies have reported low complication rates related to colonic SEMS, complications may still occur, highlighting the importance of good preparation, adequate staffing, backup systems, and informed consent [1]. Actually, due to the variations in the reported experience, we need to pay more attention to sum up the application experience, improve the success rate and reduce the complication rate, reduce the dependence on objective conditions, and avoid the radiation exposure, making it easy to carry out and promote.

The aim of this study was to explore the safety and effectiveness of SEMS insertion by colorectal surgeons using a two-person approach colonoscopy without fluoroscopic monitoring in the management of ACO especially of AMCO, based on the experience for nearly a 14-year period (2007–2020) in a tertiary referral center.

## Materials and methods

### Patients

Between October 2007 and January 2020, 434 consecutive patients (302 males and 132 females) presenting with ACO mainly from primary colorectal malignancy were considered for decompression by SEMS insertion at Chang-Hai Hospital of Naval Military Medical University.

### Treatment strategy

**Fig. 1:**
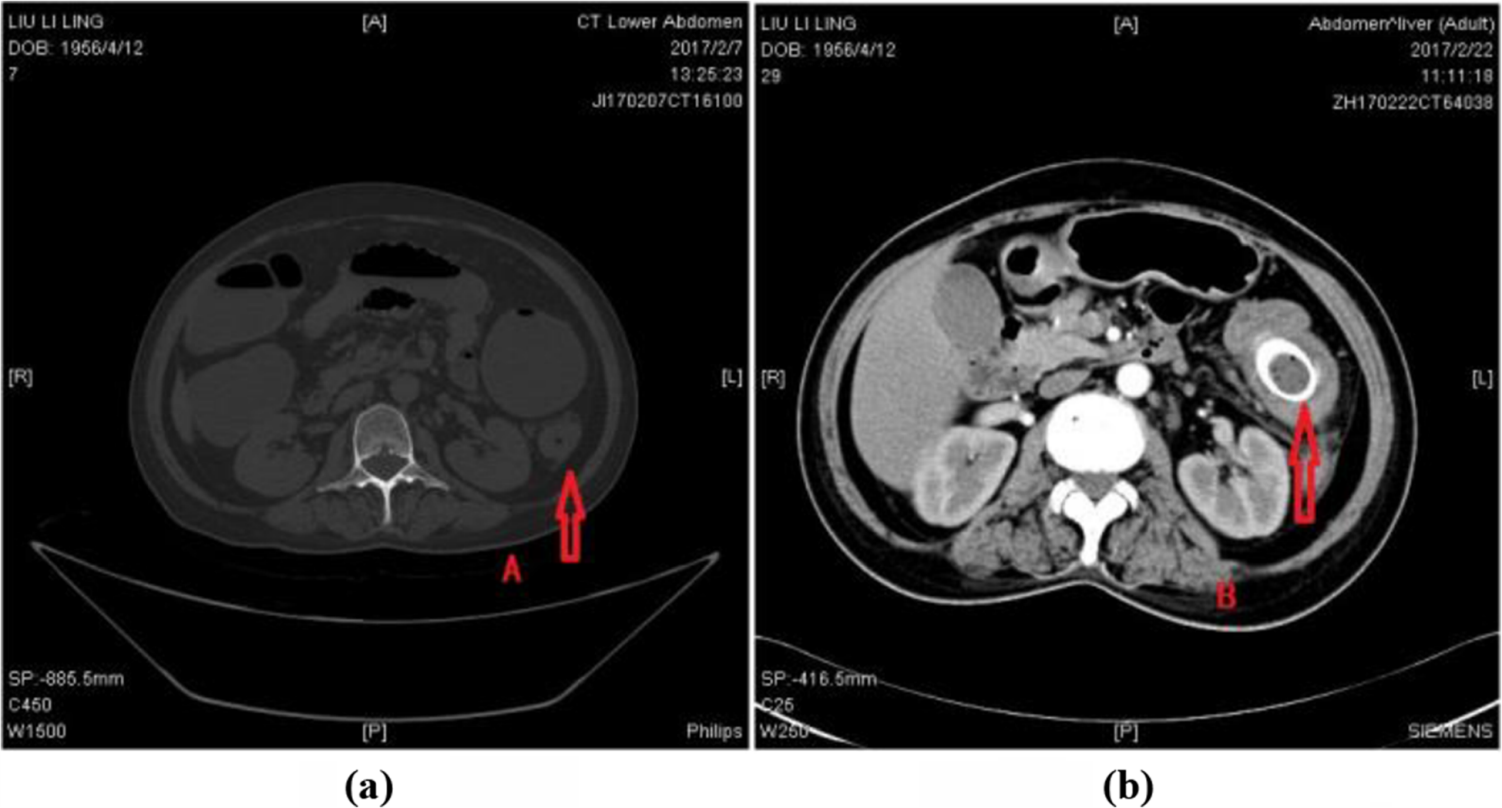
Axial computed tomography images demonstrating an obstructing carcinoma with gross proximal colonic dilatation (**a**). After successful decompression by stenting (**b**)

Since the introduction of colorectal stents to our hospital in 2007-10-11, it has almost been the colorectal surgeons’ policy to undertake this treatment strategy in all ACO patients. The ACO was defined by the presence of clinical symptoms or signs of bowel obstruction caused by colorectal-related diseases, including kinds of tumors, benign disease, and extra-luminal compression disease. Those patients who were admitted to Chang-Hai Hospital, doctors, patients, and families had a conversation about the treatment options and risks before operation. Based on the previous results and experiences, SEMS was the first option for patients with ACO in our center. We suggested patients to receive SEMS as a BTS or a palliative treatment option. However, some patients rejected our suggestions for the reasons such as the cost of treatment (in China, SEMS is not included in Healthcare Insurance), the risk of decompression failure, or the relatively longer treatment period. As a result, ES was done for some patients, and SEMS were placed as a BTS on the remaining patients. These remaining patients with ACO were included in this study. The evidence of obstruction were defined as (1) distended proximal bowel, liquid surface, transitional zone, or collapsed distal bowel on abdominal CT scans (Fig. 1), which can also demonstrate the level of obstruction, position and length of the stricture, or (2) in the colonoscopic evaluation, it is impossible to pass through the stenotic point. The clinical symptoms of obstruction were defined as (1) constipation, (2) abdominal bloating, (3) vomiting, or (4) abdominal pain [3], and the patients who had at least three of the four symptoms were enrolled.

Following SEMS insertion, a plain chest computed tomography (CT) scan, an enhanced abdominal CT scan, and an enhanced liver MRI examination were performed to complete the tumor TNM staging. The CEA and CA199 levels were measured. The biopsy of the tumor was taken for confirmation of malignant histology at the time of SEMS insertion or in 2 weeks following stent placement by a further colonoscopy. Following discussion at a multidisciplinary team (MDT) meeting or MDT outpatient clinic, patients with curable or potentially curable cancers were offered curative resection or neoadjuvant therapy.

### SEMS insertion technique

In our center, we use a two-person approach. SEMS insertions were performed using a conventional endoscope (CF-H260, Olympus, Tokyo, Japan) by experienced, qualified endoscopists without fluoroscopic monitoring. Patients underwent low-pressure, non-retention enema 3–6 times for bowel preparation and accepted insertion without sedation. Before stent insertion, the general condition of patients needs to be evaluated, and three indicators associated with tumor (the site, length, and degree of obstruction) were assessed by CT. The stent size (diameter, 18–24 mm: the 20 mm were most commonly used, 97.7%; 22 mm, 1.4%; and others, 0.9%) and length (80–170 mm: 90 mm were the most commonly used, 99.1%) were chosen according to the measured length of the obstruction on the CT images, and uncovered SEMS were mainly used. In our center, there are now two types of colonic stents manufactured in two countries available, WallFlex™ (Boston, USA) and nitinol Stents (Micro-Tech, China). The length of the stent was at least 1–2 cm longer than the stenosis at both sides to allow for adequate margins. Specific steps of operation: Inserted endoscope to the obstruction position and repeatedly washed using NS in order to expose the narrow hole of the tumor. A flexible guide wire was inserted through the endoscope channel, then passed through the obstructive lesion under endoscopic guidance without fluoroscopy [11]. Once the stent had been inserted along the guide wire across the obstruction by endoscopy through the endoscope channel, the stent was deployed through direct endoscopic guidance. After placement, the correct position and expansion of the stents were confirmed by simple abdominal radiography if necessary, rather than CT. One more stent was used in only one procedure on the day of insertion because the proximal part of the first released stent could not pass the proximal part of the tumor; in order to prevent the stent from shifting or falling off, another stent was placed inside the stent and released at the ideal position.

### Definition of outcome

**Fig. 2:**
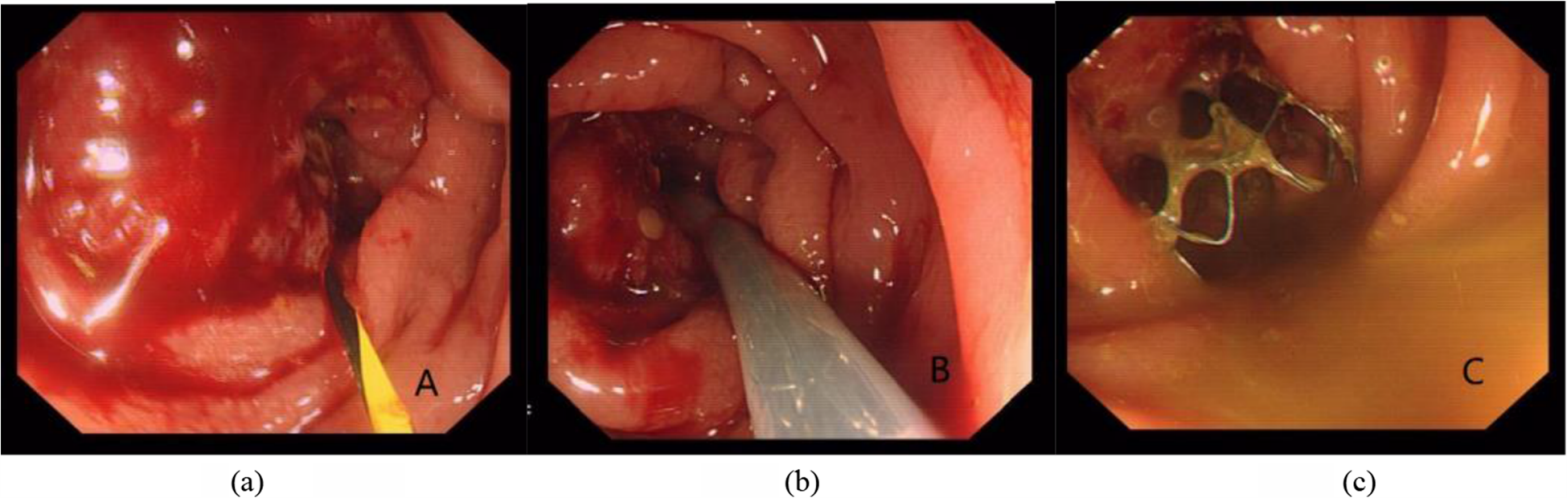
Flexible guide wire passed through the obstructive lesion (**a**). Stent was inserted along the guide wire across the obstruction (**b**). Endoscopic confirmation of appropriate stent placement, with visualization of the distal end of the stent within a normal lumen below the tumor (**c**)

**Fig. 3:**
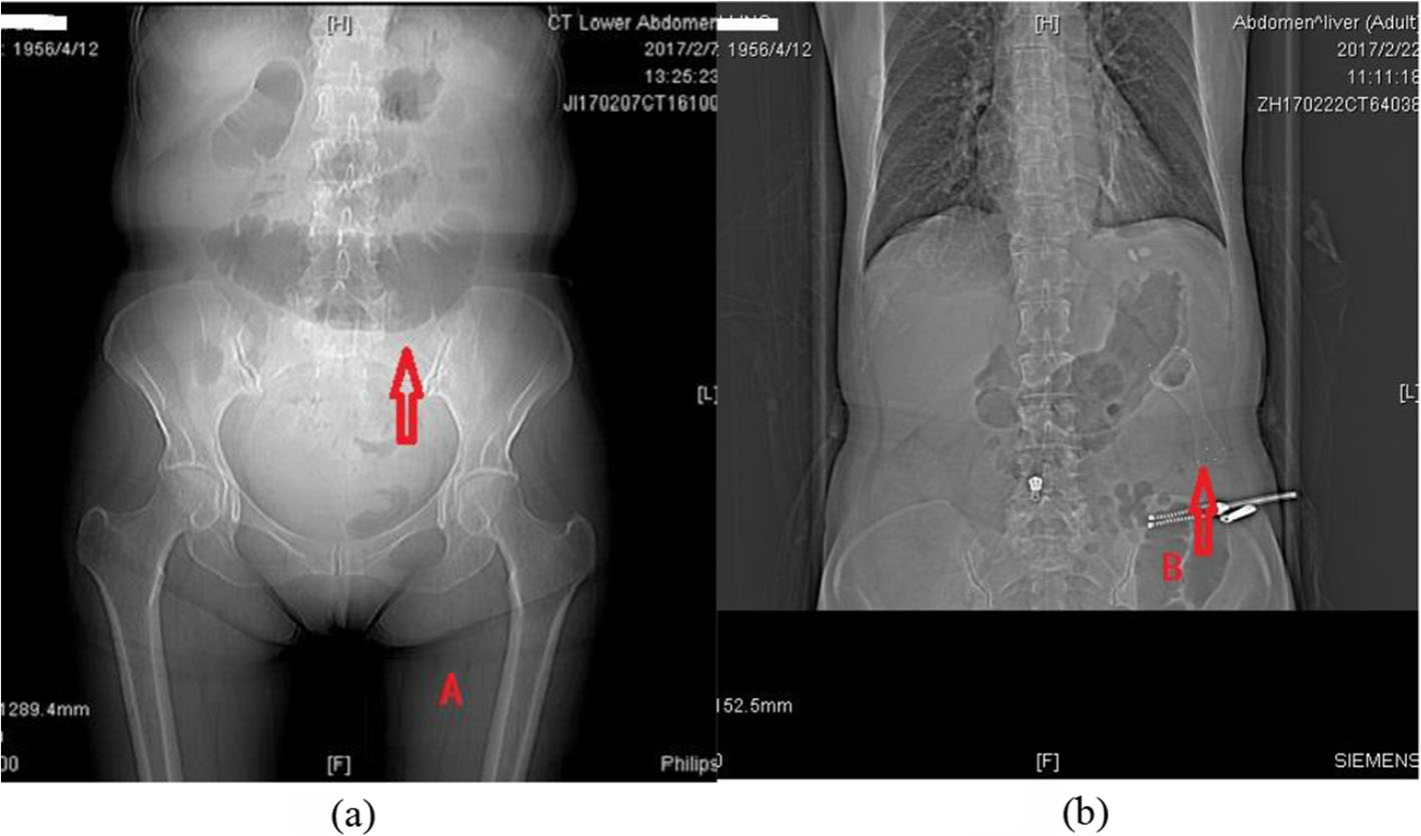
Fluoroscopic image: obstructive state before stent implantation (**a**). Depicting the correct position and the expansion of stent (**b**)

**Fig. 4:**
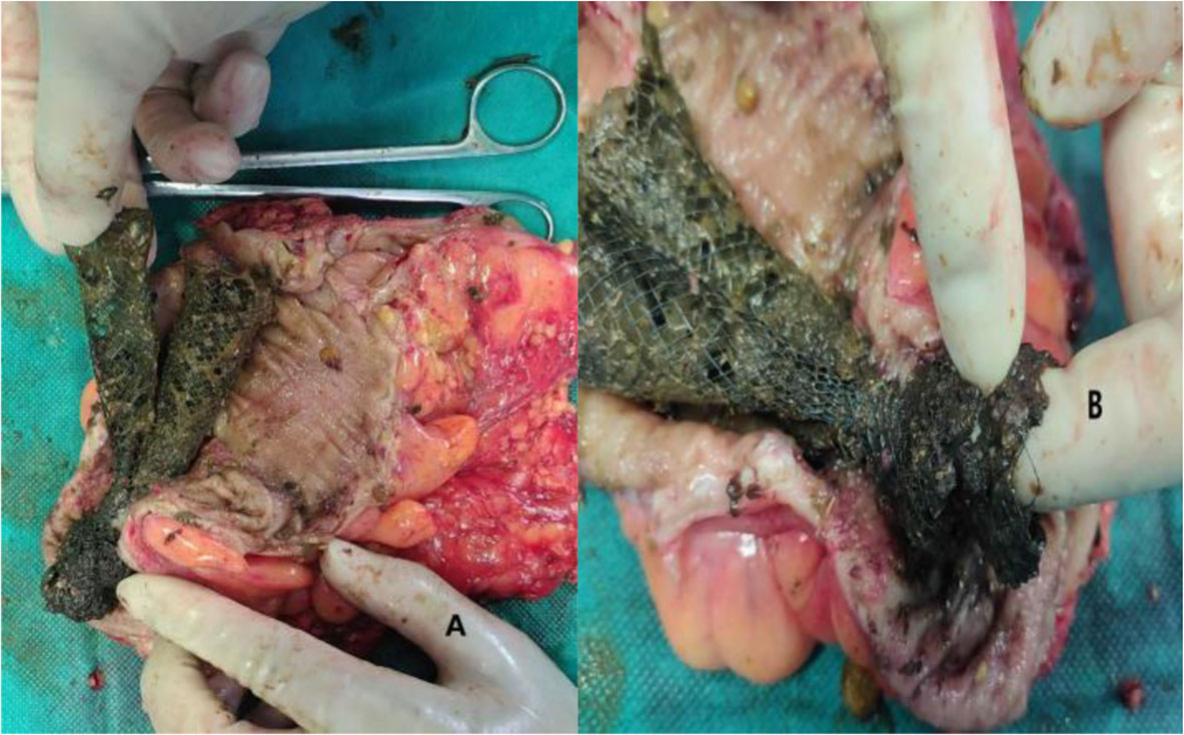
Mal-positioning result in stent occlusion-related iatrogenic acute obstruction

Technical success was defined: (1) the flexible guide wire pass through the obstructive lesion without resistance; (2) the stent should be inserted along the guide wire across the obstruction without resistance; (3) the stent should be deployed smoothly, and the distal end of the stent should be within a normal lumen below the tumor and with an appropriate margin, neither too long nor too short (Fig. 2); (4) the correct position and expansion of the stent can be confirmed by simple abdominal radiography if necessary. The proximal end of SEMS that expands like a bell is an ideal condition (Fig. 3). Clinical success was defined: decompression of the obstructed proximal bowel and restoration of luminal patency, without further interventions before the next stage of treatments, such as radical operation, neoadjuvant, and palliative treatment.

### Data retrieval and collected

We reviewed 3 data systems to confirm and identify that all SEMS patients were included in this study database, including the electronic medical record system, the endoscopy department record system, and the operating theater register system.

We collected the following data: patient demographics such as age, sex, co-morbidities, indications, site of obstruction, technical and clinical success of SEMS insertion, clinical TNM stage, length of hospital stay, details of subsequent surgery resection, and post-stenting complications including the requirement for further ES interventions.

### Statistical methods

Data were analyzed using the SPSS version 22.0 statistical software package (SPSS, Inc., Chicago, IL). Patient demographics and clinical characteristics were expressed as the mean and standard deviations, median and interquartile range, or numbers (percentages).

## Results

### Demographics and obstruction distribution

Between October 2007 and January 2020, 434 consecutive patients (302 males and 132 females) presenting with ACO mainly from primary colorectal malignancy were considered for decompression by SEMS insertion. The mean age was 63.49 ± 15.10 years. A total of 126 (29.0%) patients had co-morbidities, and some patients have two or more co-morbidities (Table 1). In this study, primary colorectal cancer (BTS and palliative) was the main study population, and its co-morbidity rate was 30.1% (121/401), which was similar to a previous report of 25.9% [3]. All patients included in this study had 31 kinds of co-morbidities, 78 cases of hypertension, 46 cases of diabetes, 15 cases of coronary heart disease, 1 case of hypokalemia, 4 cases of anemia, 6 cases of hypoproteinemia, 5 cases of stroke, 5 cases of COPD, 2 cases of asthma, 1 case of pneumoconiosis, 1 case of atrial fibrillation, 1 case of ventricular septal defect, 1 case of hyperthyroidism, 1 case of rheumatoid arthritis, 1 case of chronic interstitial nephritis, 1 case of renal failure, 1 case of pulmonary embolism, 1 case of lower extremity deep vein thrombosis, 1 case of inferior mesenteric vein embolism, 1 case of internal carotid artery stent implantation, 1 case of aortic dissection, 1 case of celiac trunk dissection, 1 case of pseudomembranous enteritis, 1 case of myasthenia gravis, 2 cases of pleural effusion, 1 case of tuberculous pleurisy,1 case of hepatitis, 3 cases of schistosomiasis cirrhosis, 1 case of portal hypertension, 1 case of obstructive jaundice, and 1 case of esophageal varices. A total of 386 patients (88.9%) had left-sided disease (tumor located at or distal to the splenic flexure). Fifteen patients (3.5%) had transverse colon disease. Thirty-three patients (7.6%) had more proximal tumors—27 at the hepatic flexure and 6 in the ascending colon (Table 2).

**Table 1:** Patient demographics (n/434, %)

Characteristic	Overall (n = 434)	BTS (n = 277, 63.8%)	Palliative stents (n = 124, 28.6%)	Others (n = 33, 7.6%)		
				Recurrent tumor (n = 10)	Benign diseases (n = 10)	Extra-luminal compression (n = 13)
**Mean age**	63.49 ± 15.10	61.26 ± 14.04	69.62 ± 16.17	58.50 ± 13.88	58.70 ± 12.28	60.15 ± 14.06
**Gender**						
Male	302, 69.6%	205, 74.0%	78, 62.9%	19, 57.6%		
Female	132, 30.4%	72, 26.0%	46, 37.1%	14, 42.4%		
**Co**-**morbidities**						
Yes	126, 29.0%	82, 29.6%	39, 31.5%	5, 15.2%		
No	308, 71.0%	195, 70.4%	85, 68.5%	28, 84.8%

**Table 2:** Overall clinic outcomes of stenting (n, %)

Characteristic	(434, %)
Overall technique success rate	428, 98.6%
Overall clinic success rate	412, 94.9%
Overall complication rate	19, 4.4%
Overall perforation rate	6, 1.4%
Overall ES required	21, 4.8%
Distribution area of obstruction	
Right hemicolon	33, 7.6%
Transverse colon	15, 3.5%
Left hemicolon	386, 88.9%

### Clinic results

The overall technique success rate was 428/434 (98.6%), and the clinic decompression rate was 412/434 (94.9%) by SEMS insertion. The overall complication rate was 19/434 (4.4%). The overall ES required was 21/434 (4.8%) (Table 2). The clinical perforation was 6/434 (1.4%); fortunately, there was no tumor seeding following SEMS placement, especially in case of perforation. Resection was done for those patients as BTS at a median of 18.5 days (14, 29 days) post-stenting, and the others were offered palliative treatment.

#### Palliative stenting

The palliation indication for SEMS was 124 patients (124/434, 28.6%). The overall technique success rate was 121/124 (97.6%), and the clinic decompression rate was 118/124 (95.2%). The overall complication rate was 4/124 (3.2%). The overall ES required was 6/124 (4.8%) (Tables 1, 3, and 4). The locations of obstruction points were ascending colon (1, 0.8%), hepatic flexure (10, 8.1%), transverse colon (7, 5.7%), splenic flexure (6, 4.8%), descending colon (18, 14.5%), descending sigmoid colon (5, 4.0%), sigmoid colon (37, 29.8%), recto-sigmoid (37, 29.8%), and rectal (23, 18.6%) (Table 3).

**Table 3:** Indications for stenting and tumor location respectively (n, %)

Location of obstruction	Indications
**BTS (277/434, 63.8%)**	
Ascending colon	5, 1.8%
Hepatic flexure	17, 6.1%
Transverse colon	6, 2.2%
Splenic flexure	22, 7.9%
Descending colon	44, 15.9%
Descending sigmoid colon	28, 10.1%
Sigmoid colon	84, 30.3%
Recto-sigmoid colon	31, 11.2%
Rectal (1NEC)	40, 14.4%
**Palliative stents (124/434, 28.6%)**	
Ascending colon	1, 0.8%
Hepatic flexure	10, 8.1%
Transverse colon	7, 5.6%
Splenic flexure	6, 4.8%
Descending colon	18, 14.5%
Descending sigmoid colon	5, 4.0%
Sigmoid colon	37, 29.8%
Recto-sigmoid	17, 13.7%
Rectal	23, 18.5%
**Others (33/434, 7.6%)**	
Recurrent tumor	10, 30.3%
Sigmoid colon	2
Rectal	8
Benign diseases	10, 30.3%
SLE	1
Anastomotic stenosis	7
Inflammatory stenosis	1
Foreign-body granuloma	1
Extra-luminal compression	13, 39.4%
Transverse colon	1
Splenic flexure	3
Descending colon	1
Descending sigmoid colon	1
Sigmoid colon	1
Rectal	6

**Table 4:** Clinic outcomes of stenting (n/N, %)

Characteristic	Palliative stents (n = 124)	BTS (n = 277)	Others (n = 33)		
			Recurrent tumor (n = 10)	Benign diseases (n = 10)	Extra-luminal compression (n = 13)
Technique success rate	121, 97.6%	274, 98.9%	10, 100%	10, 100%	13, 100%
Clinic success rate	118, 95.2%	261, 94.2%	10, 100%	10, 100%	13, 100%
Complication rate	4, 3.2%	13, 4.7%	0, 0%	2, 20%	0, 0%
ES required	6, 4.8%	15, 5.4%	0, 0%	0, 0%	0, 0%
Subsequent surgery required	7, 5.6%	258, 93.1%	2, 20%	5, 50%	2, 15.4%
Re-stenting	2, 1.6%	2, 0.7%	0, 0%	0, 0%	0, 0%

In the 3 technical failure patients, the tumor was found to be so tightly occluding the lumen of the colon that the narrow hole of the tumor could not be exposed so the guide wire could not be passed and emergency transverse colostomy was performed. The other 3 patients were a technical success but clinic failure: emergency transverse colostomy was carried out for 2 patients with incomplete decompression, and a Hartmann’s procedure was performed for another rectal tumor with sigmoid spontaneous perforation, which might be caused by the use of bevacizumab, because the perforation occurred about 24 h after stent implantation. The symptoms of intestinal obstruction were relieved, and the exhaust and defecation were restored. Intraoperative exploration showed that the perforation site was far away from the stent without a traumatic trace. Postoperative anatomy showed that the stent was in place and dilated well.

#### Stenting as a BTS

The stenting as a BTS was undertaken for 277 patients (277/434, 63.8%). The overall technique success rate was 274/277 (98.9%), and the clinic decompression rate was 261/277 (94.2%). The overall complication rate was 13/277 (4.7%). The overall ES required was 15/277 (5.4%) (Tables 1, 3, and 4), and one more stent was used in only one patient in the BTS group on the day of insertion because of the failure of decompression. The locations of obstruction points were ascending colon (5/277, 1.8%), hepatic flexure (17/277, 6.1%), transverse colon (6/277, 2.2%), splenic flexure (22/277, 7.9%), descending colon (44/277, 16.0%), descending sigmoid colon (28/277, 10.1%), sigmoid colon (84/277, 30.3%), recto-sigmoid (31/277, 11.2%), and rectal (40/277, 14.4%) (Table 3).

Fifteen of the 277 patients (5.4%) in whom stent insertion was attempted as a BTS did not have a successful decompression, although the technique success rate was higher (274/277, 98.9%) (Table 4). In the 3 technical failure patients, the tumor was found to be so tightly occluding the lumen of the colon that the narrow hole of the tumor could not be exposed, the guide wire could not be passed, emergency transverse colostomy was performed for two, and emergency right hemicolectomy was performed for another. The other 13 patients were technical success but clinic failure. In 6 patients, it was due to very tight angulation or constrictive tumor of the colon at the obstruction site precluding stent fully open post-deployment: anterior resection with primary anastomosis and defunctioning loop transverse colostomy were performed for a sigmoid tumor; emergency Hartmann’s procedure was performed for two; emergency transverse colostomy was carried out for three. In one re-stent, the decompression was satisfactory. After 200 days of neoadjuvant chemotherapy, a recto-sigmoid colon cancer patient with combined liver metastases developed incomplete obstruction 1 day before surgery; another stent was inserted to achieve decompression, but mal-positioning results in acute obstruction because of stent occlusion, so an emergency loop sigmoid colostomy was carried out under transverse abdominal fascial block anesthesia (Fig. 4). In another 5 perforation patients, emergency transverse colostomy was carried out for one; emergency Hartmann’s procedure was performed for two; subtotal colectomy was carried out for a patient with perforation after administration of avermectin, and the perforation point was far from obstruction sigmoid; anterior resection with primary anastomosis + defunctioning loop ileostomy was performed for a sigmoid tumor.

A total of 261 of the 277 patients (94.2%) in whom stent insertion was attempted as a BTS had successful decompression. Except for 4 patients who refused surgery, the remaining 257 (92.8%) patients plus 1 successful re-stenting patient in the BTS group proceeded to resection (Table 4) at a median of 18.5 days (14, 29 days) post-stenting, with no anastomotic leaks observed. The specific operation modes were right hemicolectomy (23/258, 8.9%), transverse colon resection (3/258, 1.1%), left half colon resection (83/258, 32.2%), anterior resection (132/258, 51.2%), subtotal colectomy (3/258, 1.1%), Hartmans (10/258, 3.9%), Miles (3/258, 1.1%), and stoma only (1/258, 0.4%) (Table 5).

**Table 5:** Clinic outcomes of BTS (258, %)

Characteristic	Number (n)	Stoma (n, %)	Operation method (n, %)		
			Laparoscopic assisted	Minilaparotomy	Open
Right hemicolectomy	23, 8.9%	0	5 (1 hand assisted laparoscopy)	10	8
Transverse colon resection	3, 1.1%	0	0	0	3
Left half colon resection	83, 32.2%	1	19	23	41
Anterior resection	132, 51.2%	18	27	32	73
Subtotal colectomy	3, 1.1%	2	1	0	2
Hartmann’s	10, 3.9%		3	3	4
Miles	3, 1.1%		0	0	3
Stoma	1, 0.4%	1	0	0	1
Overall	258, 100%	22, 8.5%	55, 21.3%	68, 26.4%	135, 52.3%

The total stoma rate was 8.5% (22/258, 8.5%): defunctioning loop ileostomy was performed for a patient with an abdominal abscess who accepted left half colon resection; defunctioning loop ileostomy was carried out for two who accept subtotal colectomy out of plan; palliative transverse loop colostomy was performed for a sigmoid tumor with extensive abdominal metastasis; the other eighteen patients with low or ultra-low rectal cancer accepted loop ileostomy as a routine surgery.

**Fig. 5:**
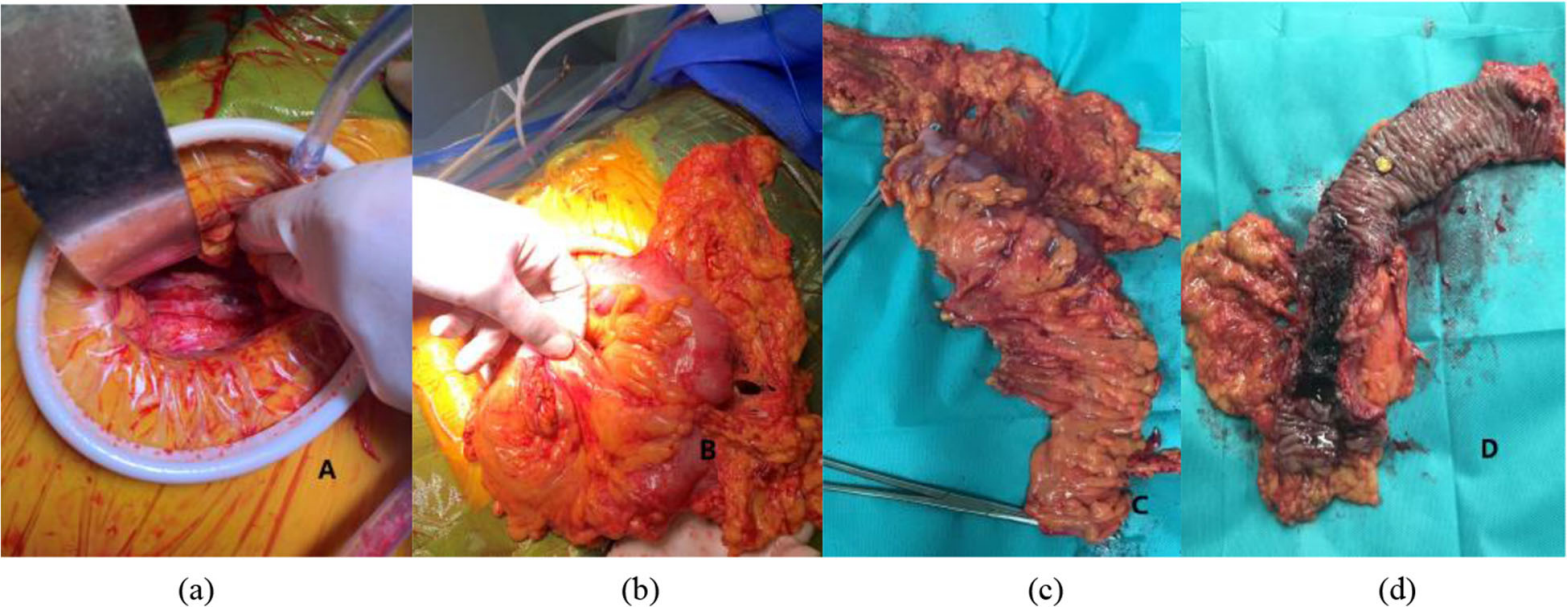
Surgical incision of left hemicolectomy by minilaparotomy operation in our center: 4–6 cm incision length (**a**). Successful decompression without intestinal wall edema (**b**). Removed bowel (**c**). Stent in the correct position (**d**)

For those 258 patients, 55 accepted laparoscopic-assisted operation (one hand-assisted laparoscopy, one Da Vinci) (55/258, 21.3%), 68 accepted minilaparotomy operation (68/258, 26.4%) (Fig. 5), and 135 accepted open operation (135/258, 52.3%), so the overall minimally invasive surgery rate was 47.7% (2007–2020) (Table 5). In fact, the rate of minimally invasive surgery in recent years is much higher than the average value from 2007 to 2020. In addition, we have carried out combined organ resection for obstructive colorectal cancer with liver metastasis after successful stent decompression since 2015. A total of 6 patients in this study had received this approach successfully.

#### Stenting for other diseases

The stent was undertaken for the other 33 patients (33/434, 7.6%) with benign diseases, recurrent tumor, and extra-luminal compression disease, etc. (Tables 1, 3, and 4). Uncover SEMS was chosen for all of these patients. Technical and clinical success was achieved in all these 33 patients as a decompression measure (33/33, 100%). The complication rate was 2/33 (6.1%). No ES was required (Table 4). The composition of the lesions leading to obstruction was recurrent tumor (10/33, 30.3%), benign diseases (10/33, 30.3%), and extra-luminal compression (13/33, 39.4%). The distribution of recurrent tumor (10/33, 30.3%) was 2 sigmoid colon and 8 rectal. The classification of benign diseases (10/33, 30.3%) was 1 SLE, 7 anastomotic stenosis, 1 inflammatory stenosis, and 1 foreign body granuloma. The distribution of extra-luminal compression (13/33, 39.4%) was 1 transverse colon, 3 splenic flexure, 1 descending colon, 1 descending sigmoid colon, 1 sigmoid colon, and 6 rectal (Tables 3 and 4). The primary lesion of extra-luminal compression (13/33, 39.4%) was 1 gastric stromal tumor, 2 pancreatic cancer, 3 retroperitoneal and pelvic tumors, 3 gastric cancer, and 4 gynecologic tumor.

### Follow-up

#### Palliative stents

**Fig. 6:**
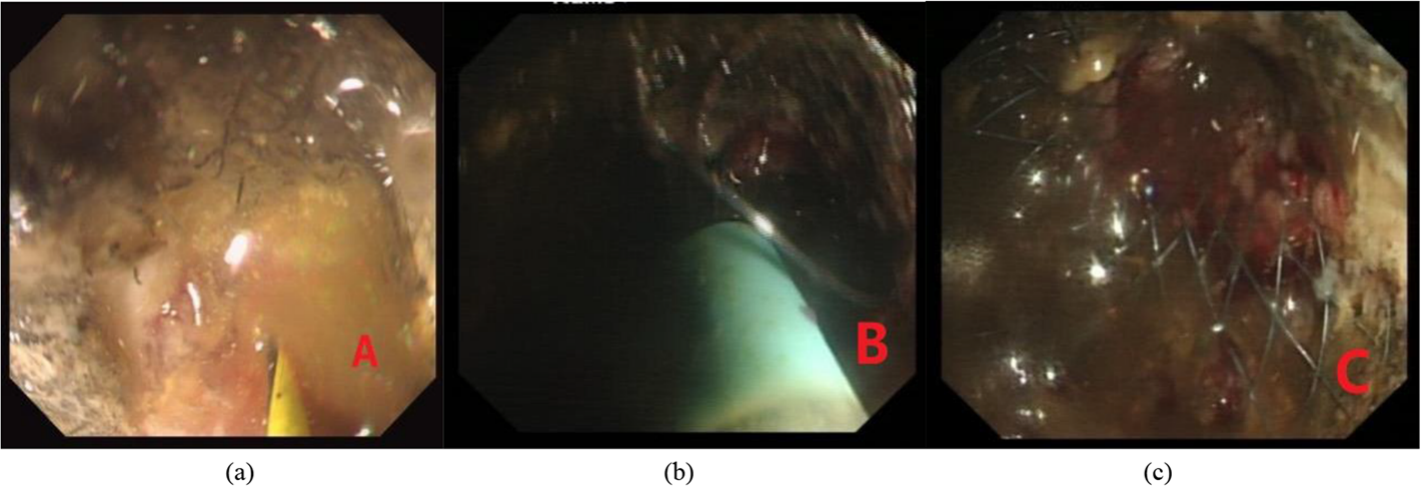
Re-stent for re-obstruction caused by local tumor ingrowth: flexible guide wire passed through the first stent (**a**). A second stent was inserted along the guide wire across the original stent (**b**). State of the deployed second stent (**c**)

Of the 124 patients who had stenting as a palliative measure, 5 subsequently required palliative transverse colostomies (3.0, 4.9, 6.2, 9.0, and 18.2 months after stenting respectively) under epidural anesthesia or laryngeal mask anesthesia and 1 subsequently required palliative sigmoid colostomy formation (86 days after stenting) under transverse abdominal fascial block due to local tumor progression and ingrowth which were unable to further endoscopic management. One patient developed small intestinal obstruction 2 months following initial successful stent placement due to extensive abdominal metastasis and made a loop ileostomy under the transverse abdominal fascial block. Thus, the overall rate of subsequent recurrent obstruction necessitating a surgical intervention in this group was 5.6% (7/124). One patient required re-stent (337 days after stenting) due to re-obstruction caused by local tumor ingrowth (Fig. 6). However, we noted one incidence of stent migration, which was solved by placing another stent, and one incidence of stent detachment without re-stent and re-obstruction.

#### Stenting for other diseases

Two patients in the recurrent tumor group accepted subsequent surgery (2/10, 20%): transverse colostomy was carried out for one and anterior resection for another, the remaining 8 patients in this group had accepted adjuvant radiotherapy or chemotherapy only. Five patients in the benign diseases group accepted subsequent surgery (5/10, 50%): three transverse colostomies and one left half colon resection were carried out for 4 anastomotic stenosis; one patient with foreign body granuloma accepted right hemicolectomy. The remaining five patients in this group had accepted medical treatment such as improve microcirculation and anti-rheumatic immunotherapy. Two patients in the extra-luminal compression group accepted subsequent surgery (2/13, 15.4%): gastro-intestinal short circuit surgery was carried out for a gastric stromal tumor; right appendectomy + greater omental excision + Hartmann’s surgery was carried out for a gynecologic tumor (Table 4). The remaining 11 patients had accepted symptomatic treatment, adjuvant radiotherapy, and chemotherapy.

#### Stenting as a BTS

Both the 30-day mortality rate and 30-day readmission rate of 258 patients in BTS were 0%. Except for common mild complications like wound infection, there were no other significant and serious postoperative complications such as anastomotic leakage, abdominal hemorrhage, and pulmonary embolism occurred.

## Discussion

### History

In 1991, the placement of SEMS in the obstructed large bowel was first described by Dohmoto [2]. For patients with incurable colorectal cancer presenting with ACO, SEMS insertion has been confirmed as a definite palliation approach [12] and is recommended by the European Society of Gastrointestinal Endoscopy (ESGE) Guideline as the preferred treatment, strong recommendation, and high-quality evidence [13], obviating more invasive surgical interventions and facilitating early administration of other treatments such as neoadjuvant radiotherapy or chemotherapy. For patients with a curable disease, SEMS placement as a BTS to avoid ES is one of the relatively recognized indications [14, 15], allowing adequate oncological staging, good colonic preparation, a quicker initiation of chemotherapy, higher elective single-stage surgical resection rate without stoma [4, 16], and the possibility of a laparoscopic approach [17], minilaparotomy, or Da Vinci radical resection. As one of the few available guidelines, ESGE recommends SEMS as a BTS to be discussed, within a shared decision-making process for potentially curable left-sided obstructive colon cancer as an alternative to ES. It should be considered carefully instead of no recommended for potentially curable proximal colon by the ESGE Guideline [13]. Furthermore, palliative management for ACO caused by extra-colonic tumors has already become one of the recognized indications of SEMS [10].

### Technique and clinic success

Concerns and controversy surrounding colonic SEMS usage mainly related to their likelihood technique and clinic success, potential complications, and efficacy. A multi-center prospective clinical study conducted by the Japan Colonic Stent Safe Procedure Research Group reported technical and clinical success rates of 97.9% and 95.5%, respectively, with a perforation rate of 2%, demonstrating that colonic SEMS can be safely inserted [18], consistent with our own data (98.6%, 94.9%, 1.4%, respectively). There were 4 patients (4/434, 0.9%) who required a second stent (1 tumor ingrowth in the palliative group, 1 migration in the palliative group, 1 failure decompression in the BTS group, 1 stool impaction in the BTS group) later.

### Complications

SEMS for managing ACO may be associated with some complications. There was a low overall complication rate (19/434, 4.4%) in our study, which might show that the procedures without fluoroscopic guidance can be feasible and safe.

#### Migration or progressive tumor in-growth

It may result in a subsequent episode of obstruction. The clinical manifestation tumor growth into the stent occurred in 6 cases (86, 148, 185, 270, 545, and 337 days after stenting) in our study and was solved by 5 palliative colostomy formation or 1 re-stenting. We undertook endoscopic stent surveillance at 3–6 monthly intervals, enabling early identification of tumor ingrowth and pay attention to whether the obstruction or incomplete obstruction symptoms occurs. Symptoms or colonoscopic findings were the indications for palliative colostomy or re-stenting before complete obstruction occurs again. We experienced 2 stent migrations, one stent migration in the BTS group occurred the day before operation and was prolapsed under endoscopy, without re-stent; another stent migration in the palliative group occurred 90 days after palliative chemotherapy with re-stent to restore patency. We experienced 3 stent migration-related detachments, two detachments in the BTS group occurred 15 days post-stenting after the intestinal wall edema subsided without re-stent, another patient with detachment in the palliative group was sensitive to palliative chemotherapy, the stent detachment 360 days after stenting without re-stent, consistent with the opinion that chemotherapy is considered a significant risk factor for stent migration [4]. So, the overall rate of migration and migration-related detachment in technique success insertions was 1.2% (5/428), less than some dislodgement and migration rate 4–10% reported [19, 20]. This was likely to be attributable to the using of CT to confirm the length of the obstruction site, chose stents longer than the obstruction, and the stents present with a “bilateral opening speaker sample” style, which may improve stent retention. This shows that the significance of CT evaluation may be far greater than that of fluoroscopy guidance. Meanwhile, stenting carried out by colorectal surgeons, using a two-person approach colonoscopy, may be more convenient for teamwork and stent deployment. This is probably the reason for the high technique success rate (428/434, 98.6%) in this study.

#### Re-obstruction

In general, when it occurs, our principle and priority of treatment are re-stenting, stoma formation, Hartmann’s, resection, and anastomosis. If surgical intervention is necessary, the priority of anesthesia methods is transverse abdominal fascial block, epidural anesthesia, laryngeal mask anesthesia, and general anesthesia. Since 2019, we began to perform loop sigmoid colostomy and loop ileostomy under transverse abdominal fascial block for patients with poor basic condition, thin, aged, and more longer sigmoid. Those work achieved surprised clinic decompression outcome avoiding the anesthesia influence on general condition.

#### Stent-related perforation

Perforation is a kind of serious complication. A recent meta-analysis by Izaskun Balciscueta et al. found that stent-related perforation is associated with an increased risk of global and locoregional recurrence [21]. Although some studies suggest that no negative effects on survival were observed for stent-related perforations [22], perforation itself is a dangerous event necessitating an ES intervention [23]. We report a 1.4% (6/434) perforation rate, which contends that a flexible guide wire should be inserted through the endoscope channel, pass through proximal to the obstructive lesion under endoscopic guidance, then the stent can insert along the guide wire across the obstruction point by endoscopy through the endoscope channel prior to the SEMS deployment. It can be considered that “the successful insertion of the guide wire is the key point of the success.” Based on the literature and our experience, we have suggestions and tips about stent-related perforation: (1) it is strictly dependent on operator expertise [4]; (2) satisfactory bowel preparation is necessary to expose the narrow hole of the tumor, then smoothly guide wire insertion can avoid mal-positioning [3]; (3) violence placement of stent can induce perforation (local or nonlocal) [4]; (4) pay more attention to inadequate colonic decompression after stenting [4]; (5) pay attention to chemotherapy especially bevacizumab [24] and radiotherapy peri-stenting [4]; (6) use carbon dioxide instead of air avoid excessive insufflations; and (7) two-person approach to colonoscopy may be more conducive to stenting [3].

#### Abdominal pain or rectal irritation symptom

There was one incidence of abdominal pain after SEMS insertion, but only for observing without special handling. Not all lesions are anatomically amenable to stenting, including those in the distal rectum that preclude deployment in normal bowel distal to the tumor [25], even if the stent is released and decompression successfully; the rectal irritation can also be very severe, and one patient chose to remove the stent for transverse colostomy because of serious rectal irritation symptom. Therefore, we always excluded rectal cancers within 6 to 8 cm of the anal verge in our center, except for very special and necessary cases.

#### Stool impaction

Six left hemicolon patients had stool impaction; one of these patients required a second stent, but decompression failed. Our effective routine treatment approach for these symptoms is 50% magnesium sulfate 50 ml plus warm saline 200 ml which was injected for retention enema through colonoscopy. From our point of view, forbidden to eat crude fiber food is the key point to prevent re-obstruction; of course, small dose of laxatives such as polyethylene glycol is necessary.

#### Thirty-day mortality and readmission

It is suggested that there is no statistically significant difference in 30-day mortality between BTS and ES group in AMCO of 5 randomized controlled trials [26] similar to the data from our center that both of the 30-day mortality rate and 30-day readmission rate of 258 patients successfully proceeded to resection in BTS were 0%. There were no significant and serious postoperative complications, consistent with a recent retrospective longitudinal cohort study using the NYS SPARCS Database which compared stenting as a BTS with ES and found that SEMS as a BTS lead to a significant quality of life advantage [27] and lower complication rate [23].

### Long-term oncological outcomes

A comparative study in our center found that there were no significant differences in terms of the long-term oncological outcomes between the SEMS group and ES group in the 3-year OS rate (55.6% versus 39.4%; P = 0.2119) and the 5-year OS rate (48.1% versus 36.4%; P = 0.3570), but with less operation time and short mean length of hospitalization in the SEMS group [3]. A retrospective study found that colon metal stents as a bridge to surgery had no significant effects on the perineural invasion [28].

### Minimally invasive surgery

Recent studies found that after SEMS placement as BTS therapy, the laparoscopic approach can be a safe alternative to ES, if the procedure is applied precociously [29]. Since the first case of laparoscopic surgery in the BTS group on 2012-08-27 in our center, we can now carry out combined organ resection for obstructive colorectal cancer patients with liver metastasis after successful stent decompression, under laparoscopic approach [30] or minimally invasive small incision [31]. The BTS group proceeded to resection at a median of 18.5 days (14, 29 days) post-stenting, with a confirmation of malignant histology by biopsy [32] and without anastomotic leak observed. It was consistent with most of the evidences and experiences that an interval of over 15 days can minimize postoperative complications. This treatment strategy was used in all patients presenting with ACO in our center instead of using anal tube decompression as reported [33].

### Special indications

#### Indication of postoperative anastomotic stenosis and extra-luminal compression disease

Postoperative strictures at the anastomotic site are reported to occur in approximately 3–30% of patients who underwent colonic resection [34], leaking due to inappropriate anastomosis at the time of reconstruction, radiation therapy, ileus, infection, and ischemia due to improper blood supply at the site might be possible causes [35]. In patients with stenosis at the lower rectum (below peritoneal reflexes), balloon dilatation under colonoscopy or anastomotic plasty transanal is the first choice in our center. In patients with stenosis located above peritoneal reflexes, balloon dilatation under colonoscopy should be chosen carefully because once the balloon water is injected too much, the violent expansion will tear the intestinal wall, resulting in perforation, which had happened in our center several years ago. For those patients, SEMS insertion should be used primarily rather than resection and anastomosis as the consequent morbidity and mortality rates owing to surgery are high. Stenting is appropriate for strictures if there are no previous radiation therapy and no postoperative anastomotic leak or if the stenosis is short and soft. It is difficult to treat anastomotic stenosis or intestinal segment stenosis caused by radiotherapy by any method mentioned above. It is reported in some studies that SEMS for extrinsic malignant colon obstruction is associated with lower technical and clinical success rates compared with intrinsic colon malignancy [36]. However, the clinical and technical success rates were 100% (13/13) in our study. Evaluating strictly and narrowing indications in our center might be the guarantee factors. Abdominal plain CT scan must be performed to confirm the lesion location and the lesion length before the procedure in order to exclude extensive metastasis and multiple site obstruction. For the obstruction caused by extraluminal compression, single-site lesions, especially the lesions infiltrating the whole intestinal wall, were considered as the best indications in our center. In addition, the clinical sensitivity of endoscopic treatment is more necessary for other benign lesions that cause ACO.

#### In extreme ACO cases, SEMS is the first choice for decompression avoiding stoma making

There were some cases in our study. One was severe systemic lupus erythematosus syndrome with poor basic condition, standard rheumatic immunotherapy was performed for this patient after stenting to relieve obstruction. One patient with coronary heart disease developed intestinal obstruction after 2 days of cardiac stent implantation. Aspirin and Plavix could not be stopped, so the risk of operation-related bleeding was high; after careful evaluation and MDT discussion, the obstruction was successfully relieved by stenting.

As mentioned above, the technique success and clinic success rate of stent do not have to be 100% always, and if the patient is not a candidate for colonic stenting or when stenting expertise is not available, it is recommended to stay in the ward and prepare for an ES decompressing stoma before attempting stent implantation, which can be the guarantee of patient safety.

### Limitations of this study

This is a retrospective study, which may have its own limitations. In the future, prospective observational studies, prospective cohort studies, and prospective randomized controlled trials are needed to explore the application value of SEMS implantation in the treatment of ACO.

## Conclusions

The clinical advantages of SEMS insertion in the management of ACO combined with little negative oncological consequences make stent an effective clinical method [4]. The usage limitation is the high perforation rate in several randomized controlled trials [23, 37, 38], other complications, and environmental health threats to operators by long-term exposure under X-ray [1]. The variation in the rates of success of SEMS insertion and associated complications reported in the literature suggests that individual expertise, institutional experience, and available resources [25] have a significant bearing on the clinical application of SEMS.

In addition to the highly recommended CT (sensitivity 96%, specificity 93%) evaluation [13], perhaps we can have the following revelation through the results of this study: (1) a two-person approach to colonoscopy may be more suitable for SEMS insertions, because good cooperation makes it more conducive to succeed; (2) it could be done without fluoroscopic monitoring, reducing the manpower cost and avoiding the radiation exposure; (3) all SEMS insertions should be carried out by colorectal surgeons, adhering to a consistent technique [23]; (4) general condition and tumor characteristic of patients need to be evaluated well, which is the foundation of success; (5) make bowel preparation well, repeatedly washed using NS in order to expose the narrow hole of the tumor is a key procedure; (6) avoid excessive air insufflations, and carbon dioxide is highly recommended; and (7) a two-person approach to colonoscopy is more conducive to the assistant of colonoscopy to assist in inserting the endoscopy and stabilizing the endoscopy body.

Operator experience has been shown to be a determinant factor to ensure appropriate stent placement and restoration of bowel function. It suggested that the adoption of this approach as standard practice only in highly specialized centers. In order to ensure the safety of stent insertion, there are recommendations. First, it has been recognized a learning curve including more than 20–30 colonic stent procedures to achieve an adequate level of technical skills needed to manage the challenging settings of emergency colonic stent insertion [39, 40]. Before entering the training, our center requires the operator to complete at least 2000 colonoscopies independently. Secondly, a tailored approach based on patient condition, surgical risk, and disease presentation seems to be the most reasonable method to define indications. In addition, emergency colonic stenting is not available in all hospitals, and protocols regarding the management of ACO should be organized to specify whether a tertiary level center can undergo this approach.

At least, stent insertion is a relatively less invasive, safe, and effective technique for colonic decompression in the setting of ACO in selected patients. It may not be applied to all situations and should be interpreted in the setting of specific clinical situations and resource availability [13], although ESGE suggests that colonic stenting should be performed with the combined use of endoscopy and fluoroscopy, but with a weak recommendation and low-quality evidence [13]. There was a low overall complication rate (19/434, 4.4%), which might show that the stent insertion without fluoroscopic guidance can be feasible, safe, and a powerful attempt to reduce the dependence on objective conditions. It can be used as palliative measures, BTS, and measures for benign diseases, recurrent tumor, and extra-luminal compression disease. Specific guidelines on the management of ACO could be useful to clarify several controversial issues in the future.

## Data Availability

The datasets generated and/or analyzed during the current study are not publicly available due to protecting individual patient privacy but are available from the corresponding authors on reasonable request.

## Abbreviations

SEMS: Self-expanding metal stent
ACO: Acute colorectal obstruction
ES: Emergency surgery
BTS: Bridge to surgery
AMCO: Acute malignant colorectal obstruction
CT: Computed tomography
MDT: Multidisciplinary team
ESGE: European Society of Gastrointestinal Endoscopy
